# Metabolic engineering of carbohydrate metabolism systems in *Corynebacterium glutamicum* for improving the efficiency of l-lysine production from mixed sugar

**DOI:** 10.1186/s12934-020-1294-7

**Published:** 2020-02-18

**Authors:** Jian-Zhong Xu, Hao-Zhe Ruan, Hai-Bo Yu, Li-Ming Liu, Weiguo Zhang

**Affiliations:** 1 The Key Laboratory of Industrial Biotechnology, Ministry of Education, School of Biotechnology, Jiangnan University, 1800# Lihu Road, Wuxi, 214122 China; 2 State Key Laboratory of Food Science and Technology, School of Biotechnology, Jiangnan University, 1800# Lihu Road, Wuxi, 214122 China

**Keywords:** l-lysine, *Corynebacterium glutamicum*, Carbohydrate metabolism systems, Fructokinase, ATP availability

## Abstract

The efficiency of industrial fermentation process mainly depends on carbon yield, final titer and productivity. To improve the efficiency of l-lysine production from mixed sugar, we engineered carbohydrate metabolism systems to enhance the effective use of sugar in this study. A functional metabolic pathway of sucrose and fructose was engineered through introduction of fructokinase from *Clostridium acetobutylicum*. l-lysine production was further increased through replacement of phosphoenolpyruvate-dependent glucose and fructose uptake system (PTS^Glc^ and PTS^Fru^) by inositol permeases (IolT1 and IolT2) and ATP-dependent glucokinase (ATP-GlK). However, the shortage of intracellular ATP has a significantly negative impact on sugar consumption rate, cell growth and l-lysine production. To overcome this defect, the recombinant strain was modified to co-express bifunctional ADP-dependent glucokinase (ADP-GlK/PFK) and NADH dehydrogenase (NDH-2) as well as to inactivate SigmaH factor (SigH), thus reducing the consumption of ATP and increasing ATP regeneration. Combination of these genetic modifications resulted in an engineered *C. glutamicum* strain K-8 capable of producing 221.3 ± 17.6 g/L l-lysine with productivity of 5.53 g/L/h and carbon yield of 0.71 g/g glucose in fed-batch fermentation. As far as we know, this is the best efficiency of l-lysine production from mixed sugar. This is also the first report for improving the efficiency of l-lysine production by systematic modification of carbohydrate metabolism systems.

## Background

l-lysine, an essential amino acids for animals and humans, is widely used as feed additive, composition of pharmaceuticals, and feedstock for cosmetics and polymer materials [1]. With the expansion and deep-going of l-lysine’s applications, the demands for l-lysine are growing rapidly in global marketplace [2]. In industry, l-lysine is mainly produced by microbial fermentation [3, 4]. Therefore, the excellent l-lysine producers and inexpensive fermentation feedstock are absolutely vital for fermentation to reduce the production costs.

*Corynebacterium glutamicum* and its subspecies are widely used as workhorse for producing l-lysine in industry [4, 5], because they possess the wide carbon resource utilization spectrum, including carbohydrate feedstock (e.g., glucose, fructose, sucrose and pentose) and non-carbohydrate feedstock (e.g., n-alkane, ethanol and organic acids) [6, 7]. In industry, the l-lysine production by *C. glutamicum* is primarily using glucose, fructose, sucrose or molasses as carbon sources, especially glucose [8]. Carbohydrate uptake and phosphorylation are mainly executed by phosphoenolpyruvate- carbohydrate phosphotransferase system (PTS) consisting of 1 membrane-bound carbohydrate-specific EIIABC component (EII) and 2 cytoplasmic components (i.e., enzyme I (EI) and histidine protein (HPr)) [9]. Previous researches have pointed out that the productivity of target products goes hand in hand with carbohydrate uptake capacity [10–12]. Although carbohydrate uptake capacity is increased with increasing the expression levels of the key genes in PTS, it will lead to overflow metabolism [10, 13]. In addition, PTS uses phosphoenolpyruvate (PEP) as phosphoryl group donor, resulting in the low intracellular PEP and the increase of flux through PEP carboxykinase [14]. It is a problem for realizing the efficient production of l-lysine that how to balance carbohydrate uptake capacity and carbohydrate consumption rate.

Aside from engineering PTS to increase the uptake rate of carbohydrates, a number of studies indicated the important role of PTS-independent carbohydrate uptake systems (i.e., non-PTS) for cell growth and production of target products (including l-lysine) by addition of *myo*-inositol to increase genes *iolT1* and *iolT2* expression level [15], by deletion or rational modification of transcriptional regulator IolR to derepress the expression of *iol*-genes [16–18], or by co-overexpression of the *myo*-inositol permease (i.e., IolT1 and IolT2; encoded by genes *iolT1* and *iolT2*, respectively) and glucokinases (e.g., GlK and PpgK; encoded by genes *glk* and *ppgK*, respectively) [19–22]. In *C. glutamicum*, the coupling system of *myo*-inositol permeases and glucokinases (designed as IGS, similarly hereinafter) has been proved to participate in carbohydrate uptake and phosphorylation [16, 17, 21–23], which belongs to non-PTS [23]. The IGS uses ATP or PolyP_n_ as phosphoryl group donor rather than PEP in *C. glutamicum*, thus increasing PEP availability [24]. In IGS, the carbohydrates are firstly transported into intracellular by *myo*-inositol permeases, and are then phosphorylated by glucokinases. However, IolT1 and IolT2 show a lower affinity for glucose as compared with EII_Glc_ [19]. Moreover, the expression of *iolT1* is repressed by IolR [18]. Lara et al. has proved that overflow metabolism can be circumvented with replacement of PTS by the coupling system of galactose permease and glucokinases in *Escherichia coli* [13]. This gives us a positive reference to construct l-lysine high-producing strain to improve the efficiency of l-lysine production.

Molasses, the major by-product of sugar industry, is a frequently-used and economical carbon source for producing many valuable fine chemicals by microbial fermentation, including ethanol [25], vitamin B_12_ [26], docosahexaenoic acid [27]. This is because that molasses contains abundant nourishments and biological active substances, such as sugars, amino acids, inorganic salts, nicotinic acid, folic acid, thiamine, etc. [7, 27, 28]. Therefore, reuse of molasses is important for sugar industry to improve economic returns. At present, molasses is one of the main feedstock for amino acids production (e.g., l-lysine [10, 29], l-glutamate [30], poly-*γ*-glutamic acid [31]), because its main component is sucrose [7]. Sucrose can be easily hydrolyzed to glucose and fructose [7]. Although molasses is wildly used for amino acid fermentation in industry, most published work focus on investigating the consumption rate of carbohydrates at single glucose, fructose, or sucrose as carbon source [32–34]. How to improve the utilization efficiency of molasses is an important problem to be solved in our present-day amino acids industry.

*Corynebacterium glutamicum* K-1 (i.e., *C. glutamicum* JL-6 ∆*pck::ppc* ∆*odx::pyc* ∆P1*gltA*/P_tac-M_*gdh*) is a l-lysine high-yielding strain with l-lysine production of 181.5 ± 7.65 g/L after cultivating 48 h in fed‑batch culture [3]. However, the productivity of *C. glutamicum* K-1 is lower as compared with the previous results (3.78 g/L/h v.s. 4.00 g/L/h) [10]. The aim of this study was to make use of mixed sugar for l-lysine production by *C. glutamicum* K-1, in which the mixture of glucose and beet molasses was designed as mixed sugar. Furthermore, in order to improve the efficiency of l-lysine production from mixed sugar, we focused on the genetic modification of genes involved in carbohydrate metabolism systems to increase the l-lysine productivity of *C. glutamicum* K-1. The results verified that carbohydrate metabolism system is a good target for improving amino acid production.

## Results and discussion

### Hetero-expression of ScrK increases l-lysine production in *C. glutamicum* K-1 from mixed sugar

**Fig. 1:**
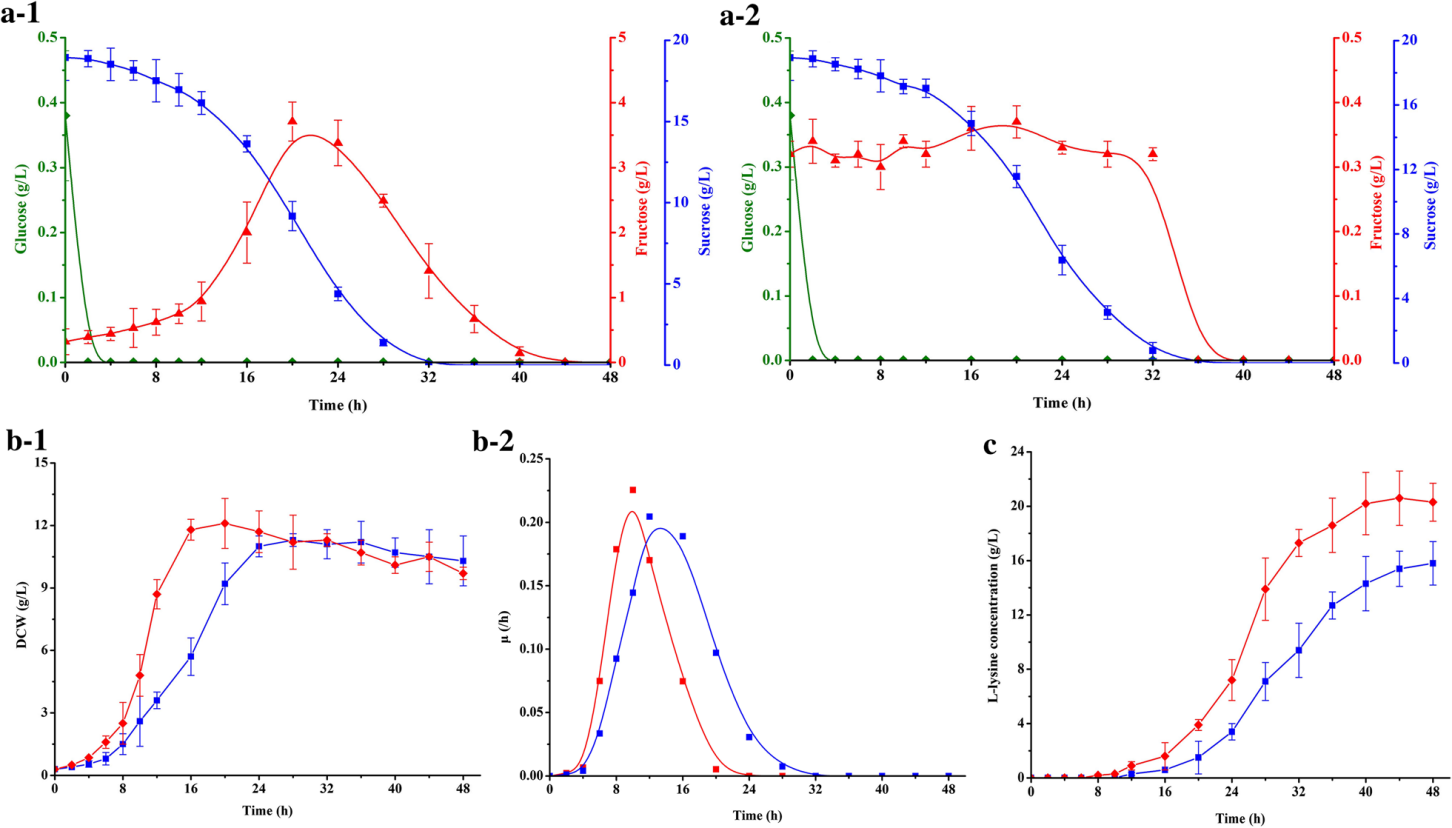
Effect of hetero-expression of fructokinase-coding gene in *C. glutamicum* on glucose (filled diamond, green lines), fructose (filled triangle, red lines) and sucrose (filled square, blue lines) consumption in CgXII^IP^-medium (**a**), and on cell growth (**b**) and l-lysine production (**c**). **a**-1 Indicates strain K-1 and **a**-2 indicates strain K-2. **b, c** Filled square and blue lines indicate strain K-1, whereas filled diamond and red lines indicate strain K-2. The data represent mean values and standard deviations obtained from two independent cultivations

According to our previous results [7], total sugar concentration in beet molasses, supplied by COFCO Biochemical (Anhui) Co., Ltd., (Anhui, China), was about 50%, and especially sucrose was the most important components (more than 47%). Sucrose was assimilated and phosphorylated by sucrose-PTS (PTS^Suc^) in *C. glutamicum* to yield sucrose-6-phosphate, and then sucrose-6-phosphate was hydrolyzed to glucose-6-phosphate and fructose by sucrose-6-phosphate hydrolase [35]. However, there is no fructokinase (ScrK) in *C. glutamicum* [36], resulting in that the intracellular fructose should be firstly excreted into extracellular and then re-assimilated via fructose-PTS (PTS^Fru^). Previous results indicated that hetero-expression of ScrK is beneficial to increase the production of NADPH-dependent products with sucrose or mixed sugar as carbon source [7, 37–39]. Therefore, hetero-expression of ScrK from *C. acetobutylicum* at *pfkB* gene loci was set as a potential strategy for enhancing l-lysine production on mixed sugar in this study. As a result, the resulted strain *C. glutsmicum* K-2 showed no fructose efflux, whereas the extracellular fructose in original strain *C. glutamicum* K-1 was increased at the initial stage of fermentation on CgXII^IP^M-medium (Fig. 1a). There was no obvious difference in DCW between *C. glutamicum* K-1 and *C. glutamicum* K-2, but the maximal specific growth rate (μ_max._) of *C. glutamicum* K-2 (μ_max._ = 0.23/h) was higher than that of *C. glutamicum* K-1 (μ_max._ = 0.20/h) (Fig. 1b). The similar results were also obtained in the previous reports [7, 39]. However, these results are contrast to the results reported by Zhang et al. [38], in which DCW of recombinant strain with hetero-expression of ScrK was 15.5% lower than that of control strain. A possible reason is that more carbon source was used to synthesize target product. Consistent with the effect on cell growth, hetero-expression of ScrK obviously increased the l-lysine production on CgXII^IP^M-medium (Fig. 1c). In addition, the sugar consumption rate of strain K-2 increased to 7.74 ± 0.45 (mmol C)/(g DCW)/h, which was 76.7% higher than that of strain K-1 (4.38 ± 0.47 (mmol C)/(g DCW)/h) (Table 1). The point is that hetero-expression of ScrK is ineffective in cell growth and l-lysine production with glucose as sole carbon source (Table 1), possibly because of the absence of fructose in culture [7].

**Table 1:** DCW, Cell growth rate, l-lysine production, and substrate consumption rate of original strain *C. glutamicum* K-1 and its derivatives in shake flask experiments

Strains	Sugar																	
	Glucose				Fructose					Sucrose					Molasses			
	DCW	μ_max._	P_Lys_	*q*_s_	DCW	μ_max._	P_Lys_	*q*_s_		DCW	μ_max._	P_Lys_		*q*_s_	DCW	μ_max._	P_Lys_	*q*_s_^a^
K-1	10.9 ± 1.2	0.25	24.1 ± 1.6	6.26 ± 0.57	8.6 ± 0.5	0.2	17.2 ± 1.8	5.13 ± 0.40		9.9 ± 0.8	0.24	18.9 ± 1.2		5.18 ± 0.32	10.3 ± 1.2	0.2	15.8 ± 2.1	4.38 ± 0.47
K-2	10.6 ± 0.5	0.25	24.3 ± 2.9	6.25 ± 0.66	8.9 ± 0.5	0.21	18.5 ± 1.7	6.06 ± 0.37		10.1 ± 0.6	0.24	23.9 ± 1.6		8.07 ± 0.51	9.8 ± 0.7	0.23	21.3 ± 2.1	7.74 ± 0.45
K-3	1.8 ± 0.2	0.06	0.5 ± 0.06	1.02 ± 0.13	2.1 ± 0.2	0.07	1.3 ± 0.3	1.21 ± 0.05		9.9 ± 1.0	0.23	24.2 ± 1.3		8.06 ± 0.54	9.8 ± 0.3	0.23	22.0 ± 1.8	7.80 ± 0.56
K-4	5.3 ± 0.7	0.12	10.4 ± 1.5	3.38 ± 0.54	9.7 ± 0.6	0.22	23.5 ± 2.2	8.05 ± 0.31		9.8 ± 1.3	0.23	24.1 ± 2.2		8.10 ± 0.72	10.0 ± 1.5	0.24	22.5 ± 1.9	7.95 ± 0.52
K-5	8.9 ± 1.0	0.18	25.3 ± 2.2	7.89 ± 0.71	10.0 ± 1.3	0.22	23.8 ± 3.0	8.06 ± 0.74		9.7 ± 0.9	0.22	24.7 ± 2.7		8.12 ± 0.53	9.8 ± 1.0	0.22	22.8 ± 2.5	7.99 ± 0.63
K-6	10.1 ± 1.4	0.23	26.0 ± 3.0	8.73 ± 0.48	10.2 ± 0.8	0.23	24.9 ± 2.5	8.21 ± 0.57		10.0 ± 1.0	0.23	25.4 ± 1.8		8.33 ± 0.75	9.8 ± 0.6	0.22	23.5 ± 1.5	8.16 ± 0.48
K-7	11.4 ± 1.2	0.26	26.4 ± 1.8	10.62 ± 0.55	10.8 ± 1.3	0.24	25.4 ± 2.1	9.75 ± 0.65		11.1 ± 1.6	0.25	26.0 ± 2.5		9.92 ± 0.49	10.7 ± 0.9	0.24	24.1 ± 2.5	9.72 ± 0.98
K-8	10.8 ± 0.7	0.24	27.6 ± 2.5	11.87 ± 0.34	10.5 ± 0.5	0.24	26.4 ± 2.8	10.92 ± 0.64		10.4 ± 0.9	0.24	27.0 ± 1.9		11.11 ± 0.62	10.5 ± 1.2	0.24	25.2 ± 1.8	10.87 ± 0.74

The culture media was CgXII^IP^-medium with 40 g/L of glucose, fructose, beet molasses or 20 g/L of sucrose as sole carbon source

DCW: Dry cell weight (g/L); μ_max.:_ The maximal specific growth rate (/h); P_Lys_: The production of l-lysine (g/L); q_s_: The substrate consumption rate (*q*_s_; mmol C/(g DCW)/h)

^a^The data was based on the sucrose consumption rate

**Fig. 2:**
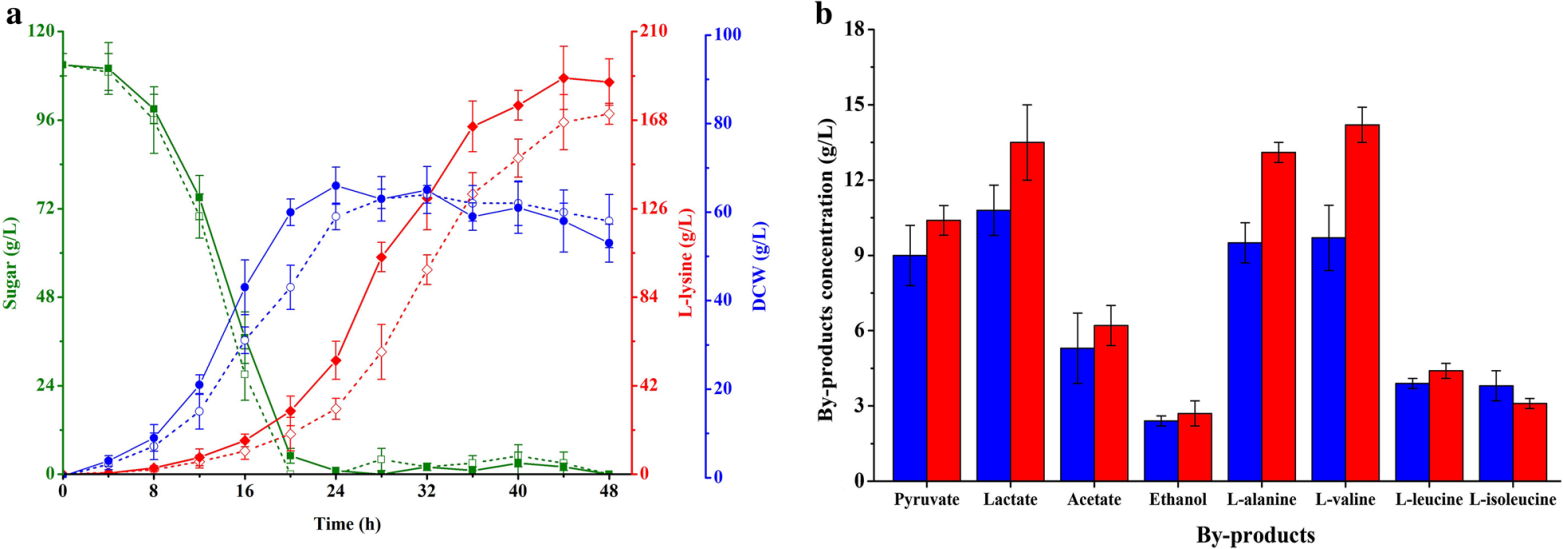
Comparison of cell growth (filled circle, blue lines), substrate consumption (filled square, green lines) and l-lysine production (filled diamond, red lines) (**a**), and by-products accumulation (**b**) of different *C. glutamicum* strains in fed-batch fermentation. Signals denote: Strain K-1 (dotted lines or blue bars) and strain K-2 (full lines or red bars). The data represent mean values and standard deviations obtained from three independent cultivations

**Fig. 3:**
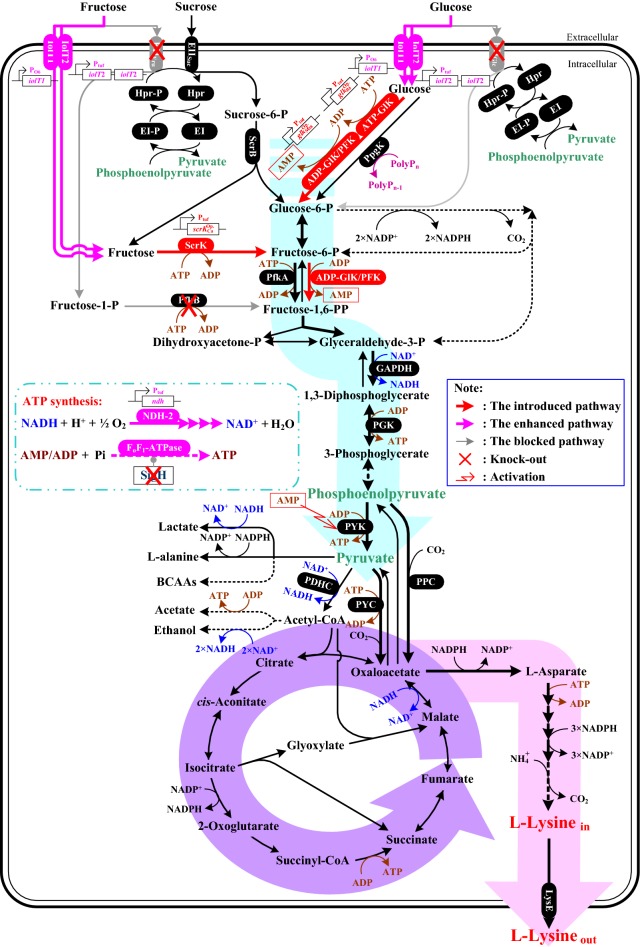
Schematic representation of l-lysine biosynthesis pathway and variant pathways for uptake of glucose, fructose and sucrose in *C. glutamicum*. Red lines indicate the introduced exogenous pathway. Pink lines indicate the strengthened endogenous pathway. “×” indicates gene deletion. *Scr*B: Sucrose-6-phosphate hydrolase, *ScrK:* Fructokinase, GlK: Glucokinase, PFK: Phosphofructokinase, GAPDH: Glyceraldehyde-3-phosphate dehydrogenase, PGK: Phosphoglycerate kinase, PYK: Pyruvate kinase, PYC: Pyruvate carboxylase, PPC: Phosphoenolpyruvate carboxylase, NDH-2: NADH dehydrogenase, ^*Op.*^ Optimized

In addition, fed-batch fermentation was carried out in a 1-L jar fermenter containing 0.25 L fermentation media to test the production performance of strain K-2. Compared with strain K-1, hetero-expression of ScrK had a trend to increase l-lysine production (Fig. 2a). The l-lysine production of strain K-2 reached to 187.3 ± 11.7 g/L, which was 9.0% higher than that of strain K-1 (171.8 ± 5.6 g/L). However, it cannot be ignored that strain K-2 accumulated large amount of by-products, especially lactate, l-alanine and l-valine (Fig. 2b). The main reason for this is that carbohydrate uptake by PTS will produce pyruvate (Fig. 3), thus leading to overflow metabolism [13]. Therefore, the next plan is to modify the carbohydrate uptake system in strain K-2 to reduce the amount of by-products.

### Deletion of *ptsG* and *ptsF* genes cause a decrease in carbohydrates consumption in *C. glutamicum*

The *ptsG* and *ptsF* genes encode membrane-bound glucose-specific EIIABC (EII_Glc_) and fructose-specific EIIABC (EII_Fru_), respectively [39]. EII_Glc_ and EII_Fru_ facilitate glucose and fructose movement across membrane, respectively (Fig. 3). Theoretically, deletion of *ptsG* and *ptsF* genes reduces the intake of sugar via PTS and thus compels strain to assimilate sugar via non-PTS. In order to verify this inference, *ptsG* and *ptsF* genes were deleted in strain K-2. However, the target strain *C. glutamicum* K-2 Δ*ptsG* Δ*ptsF* (*i.e.*, *C. glutamicum* K-3) showed bad cell growth and l-lysine production as compared with strain K-2 on CgXII^IP^-medium with glucose or fructose as sole carbon source (Table 1). This is because PTS is the major carbohydrates uptake system in *C. glutamicum* [23]. In addition, although *C. glutamicum* possess IGS for assimilating glucose and fructose, the key components (i.e., *myo*-inositol permease and glucokinases) show the low expression level and the low affinity for glucose and fructose [18, 19, 21]. Interestingly, deletion of *ptsG* and *ptsF* genes is ineffective in cell growth and l-lysine production with sucrose or molasses as carbon source (Table 1), because glucose and fructose in culture is negligible. However, it should be noted that the production performance of strain K-3 (including DCW, l-lysine production and sugar consumption rate) was dramatically disturbed by deletion of *ptsG* and *ptsF* genes in fed-batch fermentation (Additional file 1: Fig. S1). *C. glutamicum* exhibits a strong preference for glucose as carbon source [8]. And fermentation medium and feed solution mainly contain glucose (see “Methods” section) [10]. These may be the reasons for the bad production performance of strain K-3 in fed-batch fermentation. In consideration of the physiology of strain K-3 and the functional role of IGS, the expression levels of *myo*-inositol permease and glucokinases in strain K-3 should be increased to enhance the participation of IGS in carbohydrates uptake in l-lysine producer.

### Over-/hetero-expression of IolT1, IolT2, ATP-GlK and ADP-GlK accelerates carbohydrates consumption in PTS^Glc^- and PTS^Fru^-deficient strains

As mentioned above, *C. glutamicum* possess IGS for carbohydrate uptake, but *myo*-inositol permease and glucokinases show the low expression level and the low affinity for glucose and fructose [18, 19, 21]. To overcome these defects and to push carbohydrate into cell via IGS, the dominant strategy is to increase the expression level of *myo*-inositol permease and glucokinases. *myo*-inositol permease (e.g., IolT1 or IolT2) and glucokinases (e.g., PpgK or ATP-GlK) have been demonstrated to redirect carbohydrate uptake by IGS in *C. glutamicum* [19–22]. However, the expression of IolT-coding gene *iolT1* is repressed by IolR [18], and deletion of IolR-coding gene *iolR* causes certain negative effects on strain [17]. Therefore, the special promoter of *iolT1* (*i.e.*, P_o6_) with two point mutations at position -113 (A → G) and -112 (C → G) replaced the nature promoter of *iolT1* according to the reports by Brusseler et al. [17], and the nature promoter of IolT2-coding gene *iolT2* was replaced by P_*tuf*_ promoter, and then the second copy of *iolT2* gene with P_*tuf*_ promoter was introduced in strain K-3 genome, resulting in the final engineered strain *C. glutamicum* K-4. In response to these modifications, the cell growth, l-lysine production and sugar consumption rate of strain K-4 were increased by ~ 1.9 times, 19.8 times and ~ 2.3 times as compared with strain K-3 on CgXII^IP^-medium with glucose as sole carbon source, respectively (Table 1). The similar positive results were also found in fructose (increased by ~ 3.6 times, 17.1 times and ~ 5.7 times, respectively), whereas the increase of cell growth and sugar consumption rate was not obvious on sucrose and molasses (Table 1). These results indicated that IolT1 and IolT2 participate in the uptake of glucose and fructose, which were consistent with previous reports [21, 22]. Interestingly, this was also linked to an increase in the activity of PEP carboxylase (PPC) and pyruvate kinase (PYK) (Table 2). PPC catalyzes the biosynthesis of oxaloacetate from PEP, and PYK catalyzes the biosynthesis of pyruvate from PEP (Fig. 3) [1]. IGS turned away from PEP to phosphorylate carbohydrate, resulting in that a large amount of intracellular PEP can be used as substrate for PPC and PYK [14]. However, although the productivity of strain K-4 was improved as compared with strain K-3, it was lower than that of strain K-2 with glucose as sole carbon source (Table 1). Lindner et al. indicated that glucokinase must be required in PTS-independent glucose uptake system to phosphorylate glucose [24]. However, glucokinase from *C. glutamicum* shows the low affinity for glucose with *K*_m_ values of 1.0 mmol/L [40]. In addition, many studies have demonstrated that it is necessary to co-overexpression of glucokinase to get the most out of IGS [20, 22], indicating that the expression level of glucokinase-coding gene was low in *C. glutamicum*.

**Table 2:** In vitro activities of some key enzymes in genetically modified *C. glutamicum* strains and original strain *C. glutamicum* K-1 on CgXII^IP^-medium with glucose as carbon source

Strains	PTS^Glc^	GlK		PFK		ScrK	PPC	PYK	NDH-2	F_o_F_1_-ATPase
		ATP	ADP	ATP	ADP					
K-1	1.73 ± 0.27	0.046 ± 0.004	ND	0.13 ± 0.01	ND	ND	0.50 ± 0.09	1.47 ± 0.10	7.31 ± 1.52	0.39 ± 0.04
K-2	1.71 ± 0.12	0.045 ± 0.001	–	0.15 ± 0.02	–	0.11 ± 0.02	0.51 ± 0.12	1.47 ± 0.18	8.16 ± 2.03	0.44 ± 0.02
K-3	≤ 0.1	0.057 ± 0.003	–	0.02 ± 0.00	–	0.03 ± 0.02	0.17 ± 0.05	0.56 ± 0.09	2.35 ± 0.55	0.13 ± 0.02
K-4	–	0.073 ± 0.007	–	0.07 ± 0.02	–	0.07 ± 0.05	0.42 ± 0.05	1.14 ± 0.15	7.88 ± 1.64	0.41 ± 0.03
K-5	–	0.312 ± 0.018	–	0.21 ± 0.04	–	0.09 ± 0.02	0.95 ± 0.14	2.23 ± 0.21	8.43 ± 1.78	0.52 ± 0.07
K-6	–	0.325 ± 0.021	0.21 ± 0.01	0.26 ± 0.03	0.15 ± 0.03	0.11 ± 0.06	0.79 ± 0.07	2.78 ± 0.18	8.45 ± 2.25	0.53 ± 0.01
K-7	–	0.331 ± 0.019	0.21 ± 0.03	0.27 ± 0.05	0.14 ± 0.03	0.11 ± 0.03	0.80 ± 0.13	2.74 ± 0.25	26.27 ± 8.76	0.60 ± 0.08
K-8	–	0.354 ± 0.043	0.18 ± 0.04	0.32 ± 0.04	0.10 ± 0.02	0.13 ± 0.05	0.87 ± 0.09	2.59 ± 0.16	29.73 ± 5.84	0.91 ± 0.05

The unit of specific enzyme activity is U/(mg protein)

All data are meaning values of three determinations of three independent experiments with ± SD

“–” represents no test, and ND represents no detection

**Fig. 4:**
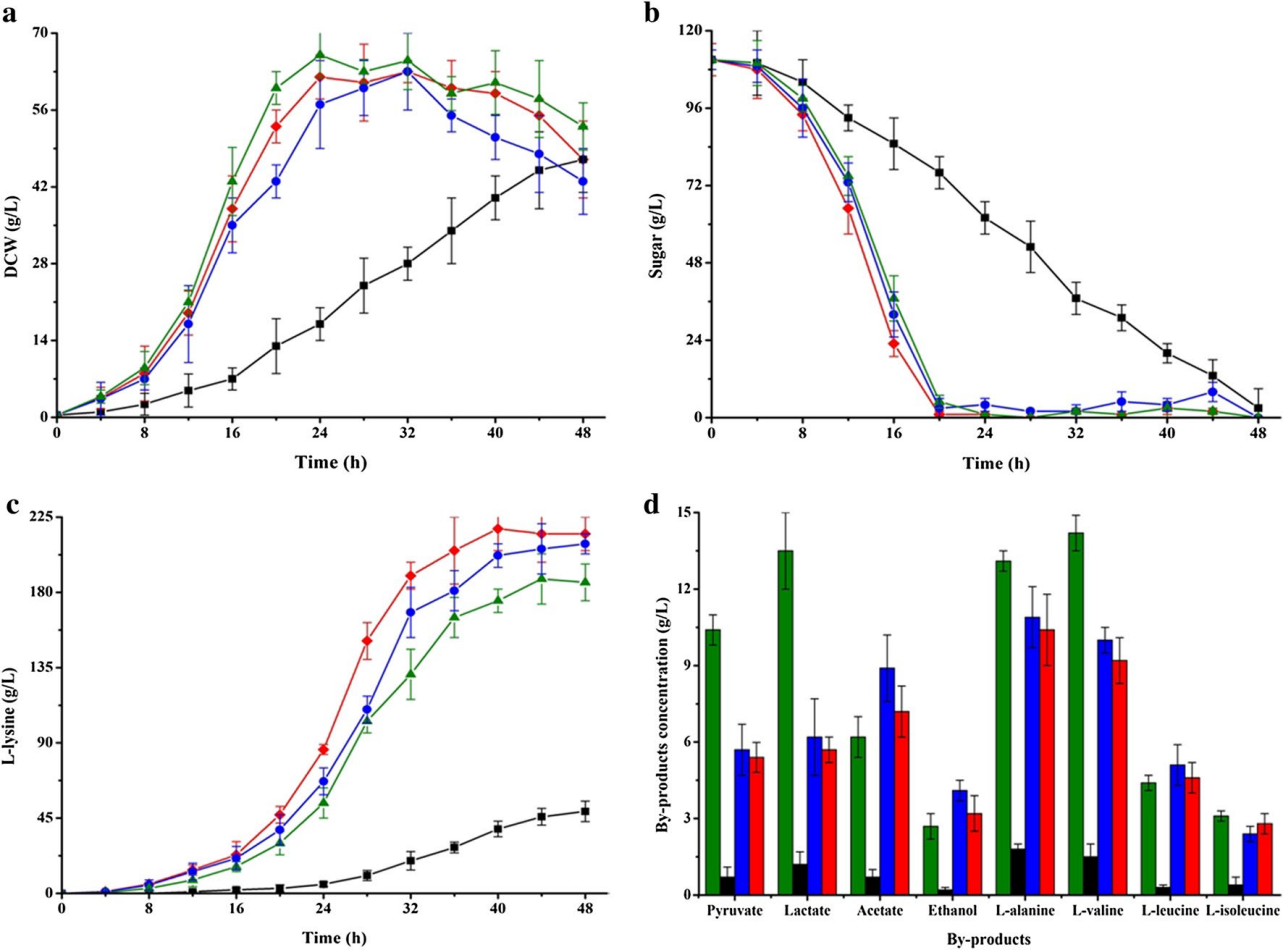
Comparison of cell growth (**a**), substrate consumption (**b**) and l-lysine production (**c**), and by-products accumulations (**d**) of strains K-2, K-4, K-5 and K-6 in fed-batch fermentation. Signals denote: Strain K-2 (filled triangle, green lines or bars), strain K-4 (filled square, black lines or bars), strain K-5 (filled circle, blue lines or bars) and strain K-6 (filled diamond, red lines or bars). The data represent mean values and standard deviations obtained from three independent cultivations

Glucokinase catalyzes the phosphorylation of glucose to glucose-6-phosphate using ATP, ADP or inorganic polyphosphates (PolyP) as phosphoryl donor [19, 41]. *C. glutamicum* has two types of glucokinases, i.e., GlK_Cg_ (ATP-dependent enzyme; ATP-GlK, encoded by *glk*_Cg_) and polyphosphate-glucose phosphotransferases (PolyP/ATP-dependent enzyme; PpgK, encoded by *ppgK*) [19], but PpgK plays a chief part in phosphorylation of glucose [40]. However, PpgK shows a low affinity for glucose with *K*_m_ values of 1.0 mmol/L [40]. In order to increase the glucose consumption of strain K-4, the native GlK_Cg_ was replaced by GlK from *Bacillus subtilis* 168 (*i.e.*, GlK_Bs_) with a high affinity for glucose (*K*_m_ = 0.24 mmol/L) [42]. In addition, the strong *tuf* promoter was located at the front of GlK_Bs_-coding gene *glk*_Bs_. The resulting strain K-5 exhibited significantly increased glucose consumption rate, cell growth and l-lysine production as compared with strain K-4 (Table 1). The final production of l-lysine by strain K-5 was 25.3 ± 2.2 g/L (0.63 g/g glucose), which is 143.3% and 4.1% higher than that of strain K-4 and K-2, respectively. This advantage was also found in the fed-batch fermentation (Fig. 4). In the fed-batch fermentation, the l-lysine production of strain K-5 reached to 209.0 ± 21.6 g/L, which was 11.6% higher than that of strain K-2 (Fig. 4c). Although most of the test by-products in strain K-5 were reduced, the accumulation of acetate and ethanol was significantly increased as compared with strain K-2 (Fig. 4d). In addition, the intracellular NADH and ATP levels were decreased in strain K-5 (Table 3). ScrK and GlK_Bs_ catalyze the phosphorylation of fructose and glucose to fructose-6-phosphate and glucose-6-phosphate using ATP as phosphoryl donor, respectively [39, 42]. Introduction of ScrK and GlK_Bs_ in strain K-5 could increase ATP consumption rate, thus perturbing the intracellular ATP balance. NADH can be oxidized to generate ATP [43], thereby meeting the demand of cell for ATP. It should be noted that the biosynthesis of acetate and ethanol involved in ATP and NADH regeneration (Fig. 3). This might be the reason why acetate and ethanol were significantly increased in strain K-5. Consistent with the previous results [44], the shortage of ATP has significantly impact on cell growth (Table 1 and Fig. 4a). To overcome this defect, the availability of ATP should be increased by reducing the consumption of ATP or/and by increasing ATP regeneration.

**Table 3:** Comparison of intracellular nucleotides concentrations in original strain and the genetically defined *C. glutamicum* strains (μmol/(g DCW))

Strains	NADH	NAD^+^		NADH/NAD^+^	NADPH	NADP^+^		NADPH/NADP^+^	ATP	
K-1	1.69 ± 0.22	7.68 ± 0.83		0.22 ± 0.03	1.71 ± 0.12	1.47 ± 0.15		1.16 ± 0.10	4.98 ± 0.42	
K-2	1.67 ± 0.23	7.59 ± 0.66		0.22 ± 0.01	1.74 ± 0.21	1.42 ± 0.19		1.23 ± 0.13	4.87 ± 0.53	
K-3	0.28 ± 0.02	2.02 ± 0.24		0.14 ± 0.02	0.25 ± 0.04	0.30 ± 007		0.82 ± 0.09	0.98 ± 0.13	
K-4	1.03 ± 0.15	5.42 ± 0.78		0.19 ± 0.03	0.99 ± 0.17	0.94 ± 0.12		1.05 ± 0.14	2.68 ± 0.19	
K-5	1.51 ± 0.18	7.57 ± 0.81		0.20 ± 0.01	1.57 ± 0.11	1.38 ± 0.15		1.14 ± 0.13	2.92 ± 0.35	
K-6	1.54 ± 0.22	7.69 ± 0.83		0.20 ± 0.02	1.48 ± 0.18	1.31 ± 0.16		1.13 ± 0.10	3.08 ± 0.32	
K-7	0.93 ± 0.23	8.58 ± 0.67		0.11 ± 0.03	1.41 ± 0.12	1.25 ± 0.13		1.13 ± 0.21	3.43 ± 0.45	
K-8	0.98 ± 0.22	8.91 ± 0.79		0.11 ± 0.01	1.16 ± 0.14	1.05 ± 0.10		1.10 ± 0.08	4.69 ± 0.47	

Exponentially growing cells cultured in CgXII^IP^ with 40 g/L glucose as sole carbon source in shake flasks were used for analysis

All data are meaning values of three determinations of three independent experiments with ± SD

Lately, ADP-dependent glucokinase (*i.e.*, ADP-GlK) was discovered in Archaea and Mammalian, which used ADP as phosphoryl donor for the phosphorylation of glucose [45, 46]. In *Methanococcus maripaludis*, a bifunctional enzyme (*i.e.*, ADP-GlK/PFK) with ability to phosphorylate glucose and fructose-6-phosphate has been reported [47]. In order to reducing the consumption of ATP, we introduced ADP-GlK/PFK from *M. maripaludis* into strain K-5. The resulting strain K-6 showed good properties in glucose consumption rate, cell growth, l-lysine production and by-products accumulation either in shake-flasks or in fed-batch fermentation as compared with strain K-5 (Table 1 and Fig. 4). Moreover, the intracellular NADH and ATP levels in strain K-6 were slightly higher than that of strain K-5 (Table 3), and this may be why strain K-6 showed the better cell growth and the lower accumulation of by-products than that of strain K-5 (Fig. 4a, d). ADP-GlK/PFK catalyzed the phosphorylation of glucose with ADP as phosphoryl donor rather than ATP [47], thus avoiding over-utilizing ATP in carbohydrate uptake. Interestingly, the activity of PYK was increased by 25% as compared with strain K-5 (Table 2). This may be because PYK is activated by AMP [48], while AMP will be synthesized from ADP in the ADP-GlK/PFK-catalyzed reactions (Fig. 3). Compared with strain K-2, however, strain K-6 exhibited the decreased intracellular NADH and ATP levels (Table 3), thereby hampering the cell growth and increasing the accumulation of acetate and ethanol (Fig. 4). The formation of acetate and ethanol involved in NADH and ATP regeneration (Fig. 3), thus making as much ATP for cell as possible.

### Overexpression of the NDH-2 benefits the further increase in carbohydrates consumption in PTS^Glc^- and PTS^Fru^-deficient strains

**Fig. 5:**
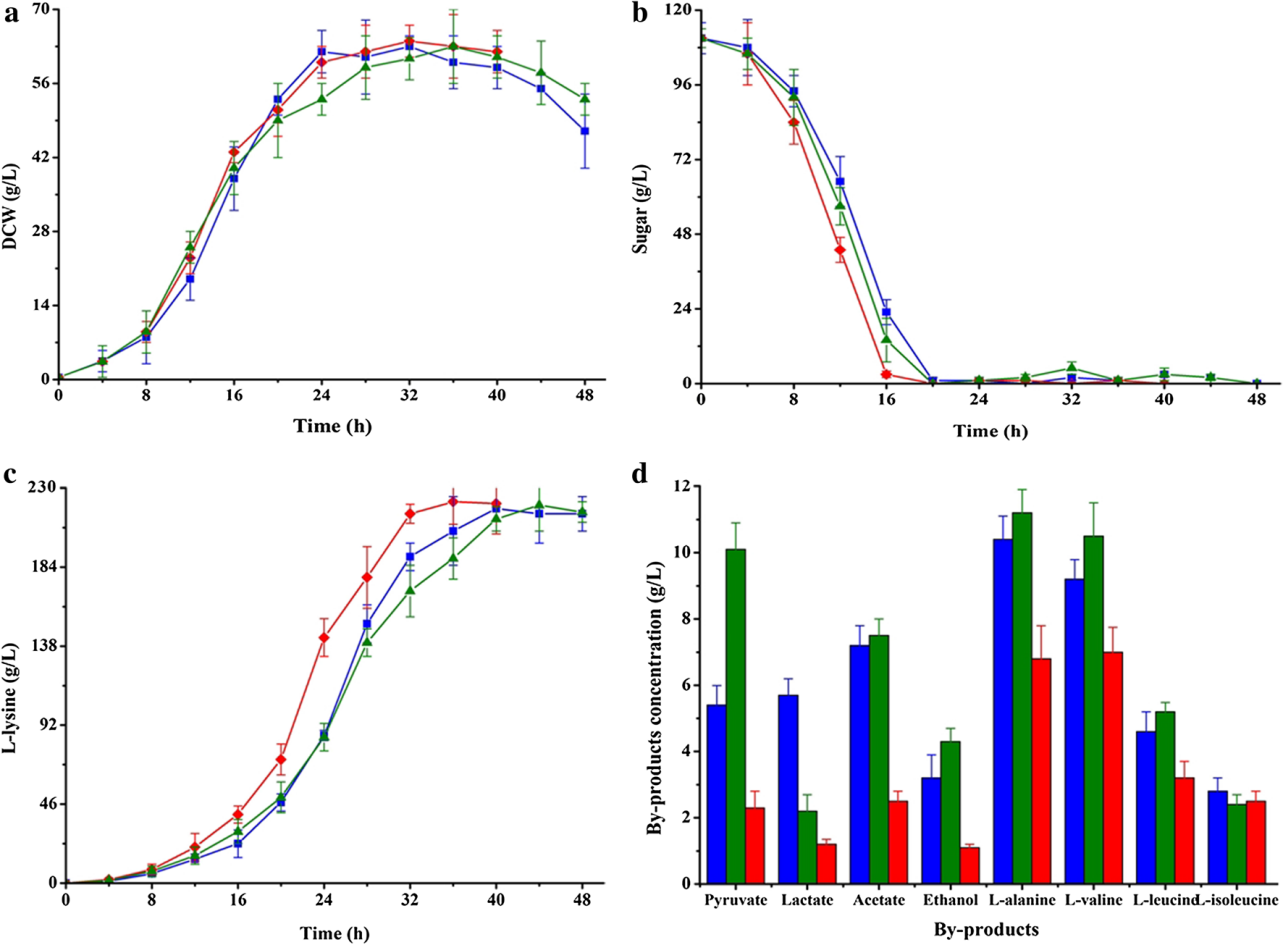
Comparison of cell growth (**a**), substrate consumption (**b**) and l-lysine production (**c**), and by-products accumulations (**d**) of strains K-6, K-7 and K-8 in fed-batch fermentation. Signals denote: Strain K-6 (filled square, blue lines or bars), strain K-7 (filled triangle, green lines or bars) and strain K-8 (filled diamond, red lines or bars). The data represent mean values and standard deviations obtained from three independent cultivations

Compared with strain K-1, the l-lysine production was drastically increased in fed-batch fermentation (from 171.8 ± 5.6 g/L to 215.2 ± 10.3 g/L), whereas the cell growth was markedly disturbed in strain K-6 because of the insufficient of ATP (Figs. 2a, 4a, c and Table 3). These results indicated that ATP may be a limiting factor for further increasing the production efficiency of l-lysine in strain K-6. ATP can be synthesized either by substrate level phosphorylation (SLP) or by electron transport phosphorylation (ETP) [44]. ETP, also known as oxidative phosphorylation, involved in the transfer of electrons from NADH to oxygen and the phosphorylation of AMP/ADP to synthesize ATP (Fig. 3) [49]. NADH dehydrogenase from *C. glutamicum* (i.e., NDH-2, encoded by *ndh* gene) is a quinone-dependent dehydrogenase, which links with the inner layer of the cytoplasmic membrane [49]. To enhance ATP synthesis by ETP, the promoter of *ndh* was substitute by the strong *tuf* promoter in this study. Consistent with the previous results [50], NADH/NAD^+^ ratio in the resulting strain *C. glutamicum* K-7 was significantly decreased as compared with strain K-6 (from 0.20 ± 0.02 to 0.11 ± 0.03; Table 3). As expected, the glucose consumption rate in shake-flasks increased from 8.73 ± 0.48 (mmol C)/(g DCW)/h in strain K-6 to 10.62 ± 0.55 (mmol C)/(g DCW)/h in strain K-7 (Table 1). In addition, the other substrates (i.e., fructose, sucrose and molasses) consumption rate was also increased during overexpression of NDH-2 in strain K-6 (Table 1). The research has shown clearly that high level of NADH/NAD^+^ ratio inhibits the activity of glyceraldehyde-3-phosphate dehydrogenase (GAPDH) and pyruvate dehydrogenase (PDHC), which are the rate-limiting enzymes in glycolysis [51]. Therefore, the effect of overexpression of NDH-2 on increasing substrate consumption rate was likely due to the increased activity of GAPDH and PDHC in *C. glutamicum*. Consistent with the previous results [52], however, the intracellular ATP levels in strain K-7 were slightly higher than that of strain K-6 (Table 3), and the l-lysine production was not obviously increased in strain K-7 (Table 1 and Fig. 5c). In contrast, strain K-7 accumulated a large amount of by-products, especially pyruvate, acetate, l-alanine and l-valine (Fig. 5d). This is presumably due to impairment of the intracellular NADH/NAD^+^ balance for l-lysine production and hence resulting in increasing the by-products biosynthetic pathway to regenerate NADH and ATP. In addition, overflow metabolism could be another reason for by-products accumulation [13]. As can be seen from Table 3, a large amount of pyruvate accumulated in the cell instead of entering into l-lysine biosynthetic pathway.

### Deletion of the *sigH* gene decreases the accumulation of by-products in *C. glutamicum* strain

Base on the chemiosmotic theory of energy coupling, the synthesis of ATP from ETP involved in energy release by transfer of electrons from NADH to oxygen and energy transfer by phosphorylation of AMP/ADP [53]. F_O_F_1_-ATPase catalyzed the formation of ATP (i.e., the phosphorylation of AMP/ADP) by proton motive force (pmf) [44]. Although F_O_F_1_-ATPase was not essential for growth of *C. glutamicum* on glucose, inactivation of F_O_F_1_-ATPase decreased the specific glucose uptake rate and mRNA levels of genes involved in amino acid biosynthesis [44]. Therefore, it is a useful strategy for increasing l-lysine production by genetically modifying F_O_F_1_-ATPase. We tried to increase the expression level of F_O_F_1_-ATPase by overexpression of F_O_F_1_-ATPase-coding gene, but it was not successful as there were eight subunits of F_O_F_1_-ATPase (encoded by *atpBEFHAGDC*) [44] (data not shown). As described by Barriuso-Iglesias et al. [54], F_O_F_1_-ATPase-coding gene was expressed at pH 9.0 because it is regulated by SigmaH factor (i.e., SigH). However, the optical pH of *C. glutamicum* in l-lysine fermentation process is neutral pH (i.e., pH ≈ 7.0). In order to increase the expression level of F_O_F_1_-ATPase-coding gene at neutral pH, we deleted SigH-coding gene *sigH* to construct a SigH-deficient strain *C. glutamicum* K-8. Consistent with previous results [54], the expression level of *atpB* was increased (increased by about 4.3 times), whereas the expression level of *sigH* was disappeared in strain K-8 as compared with strain K-7 at pH = 7 (Additional file 1: Fig. S2). Conversely, the expression level of *atpB* in strain K-8 was lower than that of strain K-7 at pH = 9 (Additional file 1: Fig. S2). These results showed that deletion of SigH increased the activity of F_O_F_1_-ATPase at neutral pH (Table 2). We also observed that the increased l-lysine production rate was induced by elevating ATP supply from ETP (Table 1 and Table 3). In other words, the more ATP is available in the cytosol, the more carbon flux will be entered into l-lysine biosynthetic pathway (Fig. 5). Previous researches have pointed out that interdiction of ATP regeneration from ETP decreased the specific glucose uptake rate in *C. glutamicum* [44, 55]. Our study again confirmed these viewpoints that strain K-8 showed the best substrate consumption rate among the test strains (Table 1). The titer of l-lysine reached 221.3 ± 17.6 g/L at a productivity of 5.53 g/L/h and a carbon yield of 0.71 g/g glucose at 40 h (Fig. 5c). Those are the highest value for l-lysine production in fed-batch fermentation (Table 4), demonstrating that this engineered *C. glutamicum* strain is a competitive platform strain for l-lysine production.

**Table 4:** Overview on the production of l-lysine by metabolic engineered *C. glutamicum*

Strains	Culturing methods	Final titers (g/L)	Productivity (g/L/h)	Yield (g/g glucose)	References
K-8	Batch	27.6	0.58	0.69	This work
	Fed-batch	221.3	5.53	0.71	This work
AM03	Batch	27.7	0.46	0.35	[60]
ZL-92	Fed-batch	201.6	5.04	0.65	[61]
JL‑69P_tac‑M_*gdh*	Fed-batch	181.5	3.78	0.65	[3]
JOV2-C7^a^	Batch	13.2	0.33	0.31	[62]
LYS-12	Fed-batch	120	4.0	0.55	[10]
MH20-22B∆leuA^a,b^	Batch	21.6	0.3	0.22	[63]

^a^Achieved in shake‑flask fermentation

^b^Estimated from reference

## Conclusions

For the first time, metabolic engineering of carbohydrate metabolism systems was identified as a critical factor for efficiently producing l-lysine from mixed sugar in *C. glutamicum*. The carbohydrate uptake system of strain was reconstructed and the intracellular ATP was complemented by enhancing ETP. We showed that hetero-expression of ScrK and introduction of optimized non-PTS were effective for increasing substrate consumption rate and l-lysine production from mixed sugar. Furthermore, substitution of the promoter of *ndh* by strong *tuf* promoter and deletion of the transcriptional regulator SigH further increased the l-lysine production and the highest substrate consumption rate, and these provided an efficient strategy for improving the efficiency of l-lysine production. The target strain K-8 produced 221.3 ± 17.6 g/L at a productivity of 5.53 g/L/h and a carbon yield of 0.71 g/g glucose in fed-batch fermentation. To the best of our knowledge, those are the highest value for l-lysine production by fed-batch fermentation in the references.

However, this yield of l-lysine in strain K-8 is still lower than the theoretical level (i.e., 0.81 g/g glucose). Thus, there is still plenty of room to enhance the yield of l-lysine. The most important by-product of strain K8 was pyruvate-family amino acids. Although adequate ATP could be used for pyruvic carboxylase (PYC) as cofactor, the activity of PYC was inhibited by l-aspartate [56]. Further improvement may be achieved by increasing the activity of PYC, for example, by overexpression of PYC-coding gene *pyc* or by site-specific mutagenesis of *pyc* to relieve feedback inhibition. Another potential strategy is to reduce the flux into biosynthetic pathway of pyruvate-family amino acids. In addition, intracellular NADPH plays an important role in l-lysine production [1]. Therefore, how to effectively improve availability of intracellular NADPH is an important problem to be solved in further improving l-lysine production strains.

## Methods

### Strains, growth medium and culture conditions

Strains used in this study are listed in Table 5. Luria–Bertani (LB) broth and LBG broth (LB supplemented with 5 g/L glucose) were used for *Escherichia coli* and *C. glutamicum*, respectively [57]. Epo medium, used for preparing electroporation-competent *C. glutamicum* cells, and LBHIS (LB, Brain Heart Infusion, and sorbitol) medium, used for obtaining recombinant strains, were prepared according to the description reported by van der Rest et al. [58]. *C. glutamicum* and *E. coli* were grown at 30 °C and 37 °C, respectively. Appropriately, strains were incubated with 50 µg/mL of kanamycin (Km), and 25 µg/mL of Km was used to obtain recombinant strains of *C. glutamicum*.

**Table 5:** Strains used in this study

Contents	Relevant characteristic(s)	References
*C. glutamicum* strains		
K-1	*C. glutamicum* JL-6 ∆*pck::ppc* ∆*odx::pyc* ∆P1*gltA*/P_tac-M_*gdh*, l-lysine-producing strain derived from strain *C. glutamicum* ATCC13032	[3]
K-2	Derivative of strain K-1 with hetero-expression of *scrK* from *C. acetobutylicum*	This work
K-3	Derivative of strain K-2 with deletion of *ptsG* and *ptsF*	This work
K-4	Derivative of strain K-3 with two point mutations in the promoter of *iolT1*, relative to the start codon at position-113 (A → G) and -112 (C → G) respectively, and with replacement of natural promoter of gene *iolT2* by *tuf* promoter as well as two copies of *iolT2* gene	This work
K-5	Derivative of strain K-4 with replacement of natural gene *glk*_Cg_ by the optimized gene *glk*_Bs_ from *B. subtilis*	This work
K-6	Derivative of strain K-5 with introduction of the optimized gene *glk*_Mm_ from *M. maripaludis*	This work
K-7	Derivative of strain K-6 with replacement of natural promoter of gene *ndh* by *tuf* promoter	This work
K-8	Derivative of strain K-7 with deletion of the gene *sigH*	This work
Plasmids		
pK18*mobsacB*	Integration vector	[59]
pK18*mobsacB*/*∆pfkB::scrK*_Ca_^Op.^	Integration vector for introduction of the cassette of *scrK* gene at *pfkB* gene loci	This work
pK18*mobsacB*/*∆ptsG*	Integration vector for deletion of *ptsG* gene	This work
pK18*mobsacB*/*∆ptsF*	Integration vector for deletion of *ptsF* gene	This work
pK18*mobsacB*/*∆ptsF::glk*_Mm_^Op.^	Integration vector for introduction of the cassette of *glk*_Mm_ gene at *ptsF* gene loci	This work
pK18*mobsacB*/*∆glk*_Cg_*::glk*_Bs_^Op.^	Integration vector for replacement of the native *glk*_Cg_ gene by the cassette of *glk*_Bs_ gene	This work
pK18*mobsacB*/P_O6_*iolT1*	Integration vector for introducing two point mutations in the promoter region of *iolT1*, relative to the start codon at position-113 (A → G) and -112 (C → G), respectively	This work
pK18*mobsacB*-P_tuf_*iolT2*	Integration vector for replacement of the nature promoter of *iolT2* gene by the *tuf* promoter	This work
pK18*mobsacB*-2 × *iolT2*	Integration vector for introducing a second copy of the *iolT2* gene with *tuf* promoter	This work
pK18*mobsacB*-P_tuf_*ndh*	Integration vector for replacement of the nature promoter of *ndh* gene by the *tuf* promoter	This work
pK18*mobsacB*/*∆sigH*	Integration vector for deletion of *sigH* gene	This work

Batch cultivation in shake flasks was carried out as described previously by Xu et al. [57]. The improved CgXII-medium (CgXII medium supplied with 0.25 g/L l-methionine and 0.6 mg/L biotin; CgXII^IP^) without carbon source was used as minimal medium for l-lysine production. The main culture was performed in triplicate using 500-mL Erlenmeyer flasks with 50 mL of CgXII^IP^-medium containing 40 g/L of glucose or 40 g/L of beet molasses, which were designed as CgXII^IP^G-medium or CgXII^IP^M-medium, respectively.

The fed-batch fermentations were carried out in a 1-L jar fermenter (BLBIO-1GC-4, Bailun Bi-Technology Co. Ltd., Shanghai, China) containing 0.25 L of medium with an inoculum size of 10% (v/v). Inoculum was obtained from a seed culture at ∆OD_562_ = 0.45 − 0.50 (at a dilution of 25-fold). The seed medium was prepared according to the description reported by Xu et al. [3]. The fermentation medium contained (per liter): 80 g glucose, 40 g beet molasses, 30 g corn steep liquor, 50 g (NH_4_)_2_SO_4_, 1.5 g KH_2_SO_4_, 1.0 g MgSO_4_∙7H_2_O, 0.02 g FeSO_4_, 0.02 g MnSO_4_, 0.5 g l-methionine, 0.05 g glycine betaine, 2.4 mg biotin (add in 4 portions), 400 μg thiamine-HCl and 2 mL antifoam. The aeration rate, pH, dissolved oxygen levels, and temperature were set as described by Becker et al. [10]. Feed solution contained (per liter) 400 g glucose, 100 g beet molasses, 40 g (NH_4_)_2_SO_4_, which prepared according to the description reported by Becker et al. [10] and was used to maintain glucose concentration at 5 ~ 10 g/L by adjusting the feeding rate according to the glucose concentration observed every 4 h. Both these media were adjusted to pH 7.0 with ammonium hydroxide.

### Construction of *C. glutamicum* recombinant strains

The plasmids and oligonucleotides used in this study are listed in Table 5 and Additional file 1: Table S1, respectively. The gene deletions and gene replacements were executed in *C. glutamicum* chromosome according to the published method [57]. The cassettes of ATP-GlK (from *B. subtilis*), ADP-GlK (from *M. maripaludis*) and fructokinase (ScrK; from *C. acetobutylicum*) with *P*_tuf_ promoter, *rrnBT1T2* terminator and *Eco*RI endonuclease was optimized for expression in *C. glutamicum* and then synthetized by GENEWIZ (Suzhou), Inc. (Suzhou, China). The plasmid construction and transformation were performed according to the previous descriptions [57]. The recombinant plasmids were transferred into *C. glutamicum* competent cell by electroporation, and the recombinant strains were screened on according to the published method [58]. The DNA manipulations and build process of the recombinant strain are stated in Additional file 1.

### Quantification of intracellular NAD^+^/NADH, NADP^+^/NADPH and ATP

The *C. glutamicum* strains were cultivated in CgXII^IP^-medium containing 40 g/L of glucose, and the logarithmic growth phase cells were used for analysis. The intracellular concentration of NAD^+^/NADH, NADP^+^/NADPH and ATP were measured using NAD^+^/NADH Quantification Colorimetric Kit, NADP^+^/NADPH Quantification Colorimetric Kit and ATP Colorimetric/Fluorometric Assay Kit (BioVision, Inc., Milpitas, CA) according to the manufacturer’s instructions, respectively.

### Analytical methods

A sample was taken from shake flasks or fermenter every 2 or 4 h. A half of sample was used to measure biomass using a spectrophotometer at 600 nm after an appropriate dilution. According to the previous description [57], the correlation factor between dry cell weight (DCW) and OD_600_ was determined as 0.318 (1 OD_600_ = 0.318 g DCW). The other half of sample was diluted 100-fold, and then used to determine glucose and l-lysine concentration using an SBA-40E immobilized enzyme biosensor (Shandong, China). l-lysine concentration was determined as lysine·HCl in duplicates. In addition, the samples were also used to determine the concentration of sucrose, fructose and by-products (including amino acids and organic acids) by high performance liquid chromatography (HPLC) according to the description of Xu et al. [57]. The enzyme activity assay is stated in Additional file 1.

## Supplementary information

**Additional file 1.** Oligonucleotides used in this study, mutation information of the genes *ptsG*, *ptsI* and *ptsH* and strategy used for construction of recombinant plasmids.

## Data Availability

All data generated or analyzed during this study are included in this published article and the additional file. The authors are willing to provide any additional data and materials related to this research that may be requested for research purposes.

## Abbreviations

IGS: The coupling system of *myo*-inositol permeases and glucokinases
CgXII^IP^-medium: The improved CgXII-medium
CgXII^IP^M-medium: CgXII^IP^-medium with 40 g/L of beet molasses
