# Supplementary material for: Metabolic engineering of carbohydrate metabolism systems in Corynebacterium glutamicum for improving the efficiency of l-lysine production from mixed sugar
Source: Microb Cell Fact. 2020 Feb 18;19:39. doi: 10.1186/s12934-020-1294-7 (PMC7029506; doi:10.1186/s12934-020-1294-7)
Supplement: Supplementary file 1 — Additional file 1. Oligonucleotides used in this study, mutation information of the genes ptsG, ptsI and ptsH and strategy used for construction of recombinant plasmids. [file 12934_2020_1294_MOESM1_ESM.docx]

**Metabolic engineering of carbohydrate metabolism systems in *Corynebacterium glutamicum* for improving the efficiency of L-lysine production from mixed sugar**

**Jian-Zhong Xu ^*^**

The Key Laboratory of Industrial Biotechnology, Ministry of Education, School of Biotechnology, Jiangnan University, 1800^#^ Lihu Road, WuXi 214122, China; E-mail: [xujianzhong@jiangnan.edu.cn](mailto:xujianzhong@jiangnan.edu.cn)

**Hao-Zhe Ruan**

The Key Laboratory of Industrial Biotechnology, Ministry of Education, School of Biotechnology, Jiangnan University, 1800^#^ Lihu Road, WuXi 214122, China; E-mail: [hz_ruan@sina.com](mailto:hz_ruan@sina.com)

**Hai-Bo Yu**

The Key Laboratory of Industrial Biotechnology, Ministry of Education, School of Biotechnology, Jiangnan University, 1800^#^ Lihu Road, WuXi 214122, China; E-mail: [aixuexi_2016@163.com](mailto:aixuexi_2016@163.com)

**Li-Ming Liu**

State Key Laboratory of Food Science and Technology, School of Biotechnology, Jiangnan University, 1800^#^ Lihu Road, WuXi 214122, China; E-mail: [liulim_jn@sina.com](mailto:liulim_jn@sina.com)

**Weiguo Zhang**

The Key Laboratory of Industrial Biotechnology, Ministry of Education, School of Biotechnology, Jiangnan University, 1800^#^ Lihu Road, WuXi 214122, China; E-mail: [zwgjnedu@sina.cn](mailto:zwgjnedu@sina.cn)

**^*^ Corresponding authors:** Jian-Zhong Xu; E-mail: [xujianzhong@jiangnan.edu.cn](mailto:xujianzhong@jiangnan.edu.cn); Tel: +86-510-85329312; Fax: +86-510-85329312

**Enzyme activity assay**

The crude enzyme was prepared according to the published method [[1](#_ENREF_1)]. After centrifugation at 4°C for 30 min at 10 000*g*, the cell-free supernatant were immediately used to determine the enzyme activities. Protein concentrations were determined using the Bradford Protein Quantification Kit according to the protocol supplied by the manufacturer (Tiangen, Beijing, China) with bovine serum albumin as standard. The analyses of enzyme activities and protein concentrations were done in triplicate. The activity of ScrK was assayed according to the methods reported by [Xu J, Zhang J, Guo Y, Zai Y and Zhang W [1]](#_ENREF_1). The analyses of ATP-GLK, ADP-GLK, and PFK were based on the protocol of [Mesak LR, Mesak FM and Dahl MK [2]](#_ENREF_2) and [Castro-Fernandez V, Bravo-Moraga F, Herrera-Morande A and Guixe V [3]](#_ENREF_3), respectively. The activities of PYK and PPC were assayed according to the methods reported by [Sawada K, Zen-in S, Wada M and Yokota A [4]](#_ENREF_4). The analyses of NDH-2 and F_o_F_1_-ATPase were based on the protocol of [Tsuge Y, Uematsu K, Yamamoto S, Suda M, Yukawa H and Inui M [5]](#_ENREF_5) and [Syroeshkin AV, Vasilyeva EA and Vinogradov AD [6]](#_ENREF_6), respectively.

**The procedures of integration vectors constructions**

**The vector pK18*mobsacB*/*∆pfkB*, pK18*mobsacB*/*∆ptsG*, pK18*mobsacB*/*∆ptsF*, pK18*mobsacB*/*∆glk*_Cg_ and pK18*mobsacB*/*∆sigH* construction**

The target gene left (i.e., *pfkB*-L, *ptsG*-L, *ptsF*-L, *glk*_Cg_-L and *sigH*-L) and right (i.e., *pfkB*-R, *ptsG*-R, *ptsF*-R, *glk*_Cg_-R and *sigH*-R) arms from *C. glutamicum* K-1 were amplified by PCR with the corresponding primer pairs, respectively. The resulting fragments were purified by the SanPrep DNA Gel Extraction Kit. The fragments of target gene left/right arms were purified and digested by suitable restriction enzyme (Table S1), respectively, and then were orderly ligated into pK18*mobsacB*, resulting in plasmid pK18*mobsacB*/*∆pfkB*, pK18*mobsacB*/*∆ptsG*, pK18*mobsacB*/*∆ptsF*, pK18*mobsacB*/*∆glk*_Cg_ and pK18*mobsacB*/*∆sigH*.

**The vectors pK18*mobsacB*/*∆pfkB::scrK*Op. Ca, pK18*mobsacB*/*∆ptsF::glk*Op. Mm and pK18*mobsacB*/*∆glk*_Cg_*::glk*OP Bs construction**

The optimized cassettes of the ATP-GlK (from *B. subtilis*)-, ADP-GlK (from *M. maripaludis*)- and fructokinase (ScrK; from *C. acetobutylicum*)-coding genes with the *P*_tuf_ promoter, *rrnBT1T2* terminator and suitable endonuclease was synthetized by GENEWIZ (Suzhou), Inc. (Suzhou, China), respectively (Table S1). DNA fragment of the optimized cassettes of the ATP-GlK, ADP-GlK and ScrK was isolated from the target plasmid after digested by suitable endonuclease. The fragment was purified and digested by suitable restriction enzyme, and then was ligated into pK18*mobsacB*/*∆pfkB,* pK18*mobsacB*/*∆ptsF* or pK18*mobsacB*/*∆glk*_Cg_ which was digested by suitable endonuclease, respectively. The resulted plasmid was designated as pK18*mobsacB*/*∆pfkB::scrK*Op. Ca, pK18*mobsacB*/*∆ptsF::glk*Op. Mm and pK18*mobsacB*/*∆glk*_Cg_*::glk*OP Bs, respectively*.*

**The integration vectors pK18*mobsacB*/P_O6_ *iolT1* construction**

The nucleotide fragment containing the 500 bases upstream and 300 bases downstream of the start codon of the gene *iolT1* with two point mutations, relative to the start codon at position -113 (A→G) and -112 (C→G) and *Sac*I endonuclease were synthetized by GENEWIZ (Suzhou), Inc. (Suzhou, China). The resulted fragments were linked to the plasmid pUC57, and the resulted plasmid was designated as pUC57/P_O6_ *iolT1.* DNA fragment of P_O6_ *iolT1* was isolated from the plasmid pUC57/P_O6_ *iolT1* after digested by *Sac*I endonuclease. The fragment was purified and digested by suitable restriction enzyme, and then was ligated into pK18*mobsacB* which was *Sac*I digested. The resulted plasmid was designated as pK18*mobsacB*/P_O6_ *iolT1*.

**The integration vectors pK18*mobsacB*-P_tuf_ *iolT2* and pK18*mobsacB*-P_tuf_ *ndh* construction**

The building process was referred to Figure S3. In PCR1, the left arm of the target gene (i.e., *iolT2*_Up_ and *ndh*_Up_) was amplified using the corresponding primer pairs, whereby an overlapping sequence with the *tuf* promoter was artificially added at the 3’-end (Table S1). In PCR2, the *tuf* promoter were amplified using the corresponding primers, whereby an overlapping sequence with the target gene (i.e., *iolT2* and *ndh*) was artificially added at the 3’-end. In PCR3, the target gene operon (i.e., *iolT2* and *ndh*) was amplified using the corresponding primer pairs. In the next step, two DNA fragments from PCR2 and PCR3 are fused in PCR4 with the *tuf* promoter and target gene operon specific primer sequences used in PCR2 and PCR3, respectively and cleaned DNA from PCR2 and PCR3. Whereafter, the DNA-fragments from PCR1 and PCR4 are fused by PCR5 using cleaned DNA from PCR1 and PCR4 as template DNA and corresponding primer pairs. Recognition sites for *Eco*RI and *Sal*I were used for vector-insert-ligation. The fragment was purified and digested by *EcoRI* and *SalI*, and then was ligated into pK18*mobsacB* which was similarly digested. The resulting plasmid was designated as pK18*mobsacB-*P_tuf_ *iolT2* and pK18*mobsacB-*P_tuf_ *ndh.*

**The integration vectors pK18*mobsacB*-2×*iolT2* construction**

The building process was referred to Figure S4. In PCR1 and PCR 2 the complete *iolT2* sequence was amplified together with flanking regions upstream and downstream of the *iolT2* gene using corresponding primer pairs, respectively (Table S1). In the next step, these two DNA fragments were fused in PCR3 using the primer pairs *iolT2*_Up_-F and *iolT2*_Down_-R, and cleaned DNA from PCR1 and PCR2, respectively as template DNA. The resulting DNA fragment contained two complete *iolT2* genes each flanked with upstream and downstream sequences as well as recognition sites for *EcoR*I and *Xba*I which were added by *iolT2*_Up_-F and *iolT2*_Down_-R, respectively. Subsequent to the amplification steps, the PCR-product was cut using restriction enzymes *EcoR*I and *Xba*I and inserted into the vector pK18*mobsacB* which was similarly digested. The resulting plasmid was designated as pK18*mobsacB*-2×*iolT2.*

**The procedures of recombinant strain construction**

**Construction of *C. glutamicum* K-1 *∆pfkB::scrK*Op. Ca (i.e., *C. glutamicum* K-2)**

The integration vector pK18*mobsacB*/*∆pfkB::scrK*Op. Ca was electro-transformed into competent *C. glutamicum* K-1, and then the resulting recombinant strain was designated *C. glutamicum* K-1 *∆pfkB::scrK*Op. Ca (i.e., *C. glutamicum* K-2).

**Construction of *C. glutamicum* K-2 *∆ptsG ∆ptsF* (i.e., *C. glutamicum* K-3)**

The integration vector pK18*mobsacB*/*∆ptsG* and pK18*mobsacB*/*∆ptsF* were orderly electro-transformed into competent *C. glutamicum* K-2, and then the resulting recombinant strain was designated *C. glutamicum* K-1 *∆ptsG ∆ptsF* (i.e., *C. glutamicum* K-3).

**Construction of *C. glutamicum* K-3 P_O6_ *iolT1* 2×P_tuf_ *iolT2* (i.e., *C. glutamicum* K-4)**

The integration vector pK18*mobsacB*/P_O6_ *iolT1* and pK18*mobsacB-*2×*iolT2* were orderly electro-transformed into competent *C. glutamicum* K-3, and then the resulting recombinant strain was designated *C. glutamicum* K-3 P_O6_ *iolT1* 2×P_tuf_ *iolT2* (i.e., *C. glutamicum* K-4).

**Construction of *C. glutamicum* K-4 *∆glk*_Cg_*::glk*Op. Bs (i.e., *C. glutamicum* K-5)**

The integration vector pK18*mobsacB*/*∆glk*_Cg_*::glk*Op. Bs was electro-transformed into competent *C. glutamicum* K-4, and then the resulting recombinant strain was designated *C. glutamicum* K-4 *∆glk*_Cg_*::glk*Op. Bs (i.e., *C. glutamicum* K-5).

**Construction of *C. glutamicum* K-5 *∆ptsF::glk*Op. Mm (i.e., *C. glutamicum* K-6)**

The integration vector pK18*mobsacB*/*∆ptsF::glk*Op. Mm was electro-transformed into competent *C. glutamicum* K-5, and then the resulting recombinant strain was designated *C. glutamicum* K-5 *∆ptsF::glk*Op. Mm (i.e., *C. glutamicum* K-6).

**Construction of *C. glutamicum* K-6 P_tuf_ *ndh* (i.e., *C. glutamicum* K-7)**

The integration vector pK18*mobsacB-*P_tuf_ *ndh* was electro-transformed into competent *C. glutamicum* K-6, and then the resulting recombinant strain was designated *C. glutamicum* K-6 P_tuf_ *ndh* (i.e., *C. glutamicum* K-7).

**Construction of *C. glutamicum* K-7 *∆sigH* (i.e., *C. glutamicum* K-8)**

The integration vector pK18*mobsacB*/*∆sigH* was electro-transformed into competent *C. glutamicum* K-7, and then the resulting recombinant strain was designated *C. glutamicum* K-7 *∆sigH* (i.e., *C. glutamicum* K-8).

**Table S1** The oligonucleotides used in this study

| Oligonucleotide | | 5’→3’ sequence *^a^* | Cleavage sites | Purposes |
| --- | --- | --- | --- | --- |
| *ptsG*-L-F | | CCCAAGCTTGATACCTGGAATCATTCG | *Hin*dIII | PCR for the *ptsG* left arm, *ptsG*-L |
| *ptsG*-L-R | | TCCCCCCGGGCAGTGAGGCACCAAGCAG | *Xma*I |  |
| *ptsG*-R-F | | TCCCCCCGGGGCTGCAACCGCCGTGGCAGC | *Xma*I | PCR for *ptsG* right arm, *ptsG*-R |
| *ptsG*-R-R | | CGGAATTCAGGTCCATCTTGCGGTCAAG | *Eco*RI |  |
| *ptsF*-L-F | | GCTCTAGAGGAGCGGTGTTGGTCAACGC | *Xba*I | PCR for the *ptsF* left arm, *ptsF*-L |
| *ptsF*-L-R | | TCCCCCCGGGGCATGCGGTGATTGCCACG | *Xma*I |  |
| *ptsF*-R-F | | TCCCCCCGGGTCATGATGTGTTTCGACCTCG | *Xma*I | PCR for *ptsF* right arm, *ptsF*-R |
| *ptsF*-R-R | | CGGAATTCCTCTGCGCCCAGCGCCATGATC | *Eco*RI |  |
| *pfkB*-L-F | | GCTCTAGAGAGCCCATGGGTACGTCTC | *Xba*I | PCR for the *pfkB* left arm, *pfkB*-L |
| *pfkB*-L-R | | TCCCCCCGGGGCCGGCTGGGAACACAGC | *Xma*I |  |
| *pfkB*-R-F | | TCCCCCCGGGGGAGCGGTGTTGGTCAACGC | *Xma*I | PCR for *pfkB* right arm, *pfkB*-R |
| *pfkB*-R-R | | CGGAATTCTGTGTGCGATACCGGTTG | *Eco*RI |  |
| *sigH*-L-F | | AACTGCAGGAGTAGGGAAGTGCCTGGTG | *Pst*I | PCR for the *sigH* left arm, *sigH*-L |
| *sigH*-L-R | | ACGCGTCGACCTAGTCATGCGCAGAGCAC | *Sal*I |  |
| *sigH*-R-F | | ACGCGTCGACACTCGGAACTGTGATGTC | *Sal*I | PCR for *sigH* right arm, *sigH*-R |
| *sigH*-R-R | | GCTCTAGATATCGAAATGGATTGGCGC | *Xba*I |  |
| *iolT2-*F | **ATGACGGACATCAAGGCCACATCAAGTACATCG** | | *-* | PCR for the 900 bases upstream of *iolT2* operon |
| *iolT2*-R | CCCAAGCTTGAGCAGACGAACCAGCCAT | | *Hin*dIII |  |
| *iolT2*_Up_-F | CGGAATTCTTGCGCACGCAGTTTCCTTCAACG | | *Eco*RI | PCR for the upstream of promoter of *iolT2*, *iolT2*_Up_ |
| *iolT2*_Up_-P_tuf_-R | **CACTTACCCTACGCGCCTACTGACACGCT**AACAACCGGGAAGGTGGCCG | | *-* |  |
| P*tuf_iolT2_*-F | **AGCGTGTCAGTAGGCGCGTAGGGTAAGTG** | | *-* | PCR for the *tuf* promoter, P_tuf_ used for *iolT2* modification |
| P*tuf_iolT2_*-R | **CGATGTACTTGATGTGGCCTTGATGTCCGTCAT**TGTATGTCCTCCTGGAG | | *-* |  |
| *iolT2*_Up_-F | CGGAATTCCGATCACGCAGCATTCACCGGCCAG | | *Eco*RI | PCR for the *iolT2* operon with its upstream |
| *iolT2*_Mind_-R | **CCGGTCGACCCCACATGTCGCGGATCCGGCG**TTAAGCCTTCTTGAAGATCTG | | *-* |  |
| *iolT2*_Mind_-F | **CGCCGGATCCGCGACATGTGGGGTCGACCGG**ATGACGGACATCAAGGC | | *-* | PCR for the *iolT2* operon with its downstream |
| *iolT2*_Down_-R | GCTCTAGATCCACTGGAAGCGACATGGTG | | *Xba*I |  |
| *ndh-*F | **ATGTCAGTTAACCCAACCCGCCCCGAAGGCGGCCGTCA** | | *-* | PCR for the 900 bases upstream of *ndh* operon |
| *ndh*-R | CCCAAGCTTGCGGCCTGCGCGGTCGGTCTCAAC | | *Hin*dIII |  |
| *ndh*_Up_-F | CGGAATTCCTATTGCGACCAGCTCCCGGCACATG | | *Eco*RI | PCR for the upstream of promoter of *ndh*, *ndh*_Up_ |
| *ndh*_Up_-P_tuf_-R | **CACTTACCCTACGCGCCTACTGACACGCT**GCACGTGTACGCGTTAAACAATAG | | *-* |  |
| P*tuf_ndh_*-F | **AGCGTGTCAGTAGGCGCGTAGGGTAAGTG** | | *-* | PCR for the *tuf* promoter, P_tuf_ used for *ndh* modification |
| P*tuf_ndh_*-R | **TGACGGCCGCCTTCGGGGCGGGTTGGGTTAACTGACAT**TGTATGTCCTCCTGGAG | | *-* |  |

*^a^* Cleavage sites are underlined; Sequence in bold: homologous recombination sequences.

-: No cleavage sites.


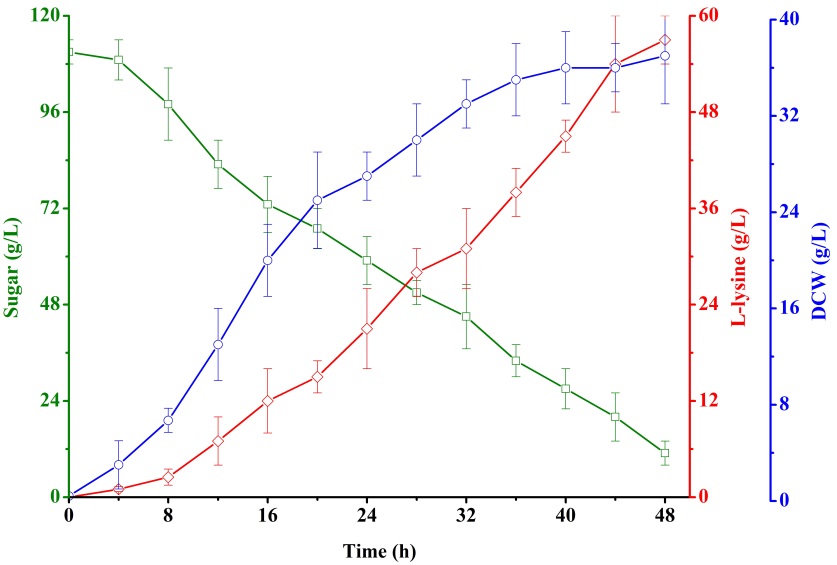


**Figure S1.** Time course of L-lysine fed-batch fermentations of strains *C. glutamicum* K-3 in 5-L fermentors. Fed-batch cultivation was performed with an initial glucose concentration of 80 g L^-1^. Signals denote: DCW (circle, blue), Glucose (square, green), and L-lysine (diamond, red). The data represent mean values and standard deviations obtained from two independent cultivations.

**Figure S2.** Analyzing the expression levels of *sigH* and *atpB* from strain K-7 and strain K-8 between at pH 7 and pH 9 by semiquantitative RT-PCR. The numerical value in Figures represents fold change of gene expression level between different conditions.

**Figure S3.** Strategy used for construction of recombinant plasmid used for the replacement of promoter of the target gene. *T* represents the modified gene; *T_Up_* represents the upstream regions of the modified gene; *P* represents the promoter; *P1-P6* represents the primers; *E1* and *E2* represent the restriction enzymes. The lines in the same color represent the homologous sequence.

**Figure S4.** Strategy used for construction of recombinant plasmid pK18*mobsacB*-2×*iolT2*. *Ren box* represents the promoter *P*_tuf_; *Gray and black boxes* represent the upstream and downstream of *iolT2* gene, respectively; *P1-P4* represents the primers; *E1* and *E2* represent the restriction enzymes. The lines in the same color represent the homologous sequence.

**Supplementary References**

1. Xu J, Zhang J, Guo Y, Zai Y, Zhang W: **Improvement of cell growth and L-lysine production by genetically modified *Corynebacterium glutamicum* during growth on molasses.** *J Ind Microbiol Biotechnol* 2013, **40:**1423-1432.

2. Mesak LR, Mesak FM, Dahl MK: ***Bacillus subtilis* GlcK activity requires cysteines within a motif that discriminates microbial glucokinases into two lineages.** *BMC Microbiol* 2004, **4:**6.

3. Castro-Fernandez V, Bravo-Moraga F, Herrera-Morande A, Guixe V: **Bifunctional ADP-dependent phosphofructokinase/ glucokinase activity in the order Methanococcales biochemical characterization of the mesophilic enzyme from *Methanococcus maripaludis*.** *FEBS Journal* 2014, **281:**2017-2029.

4. Sawada K, Zen-in S, Wada M, Yokota A: **Metabolic changes in a pyruvate kinase gene deletion mutant of *Corynebacterium glutamicum* ATCC 13032.** *Metab Eng* 2010, **12:**401-407.

5. Tsuge Y, Uematsu K, Yamamoto S, Suda M, Yukawa H, Inui M: **Glucose consumption rate critically depends on redox state in *Corynebacterium glutamicum* under oxygen deprivation.** *Appl Microbiol Biotechnol* 2015, **99:**5573-5582.

6. Syroeshkin AV, Vasilyeva EA, Vinogradov AD: **ATP ssynthesis catalyzed by the mitochondrial F_1_F_o_ ATP synthase is not a reversal of its ATP activity.** *FEEBS Letters* 1995, **370:**281-281.
